# Intra-Capsular Fracture of Neck of Femur

**Published:** 1876-05

**Authors:** T. J. Maxwell

**Affiliations:** Olena, Illinois


### INTRA-CAPSULAR FRACTURE OF NECK OF FEMUR.

BY T. J. MAXWELL, M.D., OLENA, ILLINOIS.

(Read before the Military Tract Medical Society, Jan. 11, 1876, at Galesburg, Ill.)

I wish to lay before this Society a single clinical observation upon a subject that Sir Astley Cooper put to rest more than half a century ago. I refer to the subject of fracture of the neck of the thigh bone within the capsular ligament. As my case, in its treatment and results, conflicts with the teaching and experience of the great master, I approach the subject with the most profound respect for his genius and great learning, and hope you will not think me presumptuous.

Sir Astley doomed every man and woman who should be so unfortunate as to suffer from a fracture within the capsular ligament of the femur to a life of halting and deformity. Indeed, such was his experience without exception, and such has been generally that of the profession since his time.

With these prefatory remarks, I proceed to give the history of a case that came under my observation, the method of treatment, and its results, together with some remarks on the subject.

Mrs. J. G——, æt. 50, on the morning of Jan. 14, 1871, slipped and fell upon the hard, icy ground while attempting to go up a short inclined plane, striking on the right hip. She said she felt somewhat stunned, but did not suspect any serious injury until she attempted to rise, and then discovered that she was unable to use her right leg. She immediately called for assistance, and was carried into the house and laid on a pallet on the floor, where I found her about one hour after the accident.

On examination, I found the foot of the injured side everted, lying on its outer edge. Free motion of the limb in every direction, rotation and flexion of the thigh upon the pelvis, could be accomplished without trouble or the application of much force, though with considerable pain to the patient. By measuring the limb from the anterior superior spinous process of the ilium to the internal malleolus, it was discovered that the injured leg was fully one inch shorter than the other.

The leg could easily be extended to its full length, and on rotating a little in that position crepitation was developed. The lady was of thin, spare habit, and afforded an excellent opportunity for free examination about the articulation. I could grasp the trochanter major in its entire extent to the neck of the bone, and follow all its movements. There was but little swelling, and a moderate degree of pain on handling. One marked feature of the appearance and feel of the hip, was the flattening in the region of the trochanter major, which was in no way improved by extending the leg to its full length. Grasping the trochanter and rotating the leg showed that it did not describe the arc of a circle like the uninjured one, but rotated on its own axis, or nearly so. I applied Day's splint for fractures of the thigh. Had no trouble in getting the leg to its normal length; but that flattened condition of the trochanter was unchanged, and she complained of pain and uneasiness in the region of the hip and groin constantly.

I continued this appliance for ten days, and then determined to change, as it did not accomplish the purpose, that is, to keep the parts in coaptation.

Accordingly, the splint was removed, and the following treatment substituted: A pulley was fixed to the foot of the bed, and another to the side about the centre. The foot was elevated about nine inches on the front and about six on the back, and the front side of the head about three, so that the tendency of a person lying on the bed would be to slip to the head and back part. The patient was then placed in bed, and extension made by means of a weight attached to a cord passing over the pulley at the foot of the bed, and fastened to the ends of the adhesive straps which extended beyond the heel.

These adhesive straps were applied to the whole length of the thigh and leg, and, of course, covered by a bandage from the toes to the body. The lateral extending bandage, about four inches wide, was then passed around the thigh close to the body, and fastened to a cord which was carried over the pulley at the side of the bed, to which was suspended a tin bucket for weights. Weights were gradually added to the bucket at the foot until the leg was brought and kept at its normal length, and the lateral extension was increased, until the trochanter major could be grasped by the hand and felt to be as prominent as that on the well side.

The weight required to accomplish extension was about twenty pounds. For lateral extension—to lift the trochanter from its position against the posterior lip of the acetabulum—about twelve or fourteen pounds.

As soon as the lower fragment was brought out to its proper position, all pain ceased, and was not afterwards complained of during the long term of confinement.

She remained in bed with this apparatus until the fourteenth day of March, just two months to a day from the date of injury. When it was removed, motion and adhesion of the part appeared perfect. The adhesive straps, by which extension was secured, caused some slight abrasions of the skin; the knee joint was swollen and painful, and it was several weeks before it resumed its function so as to be useful.

The leg maintained its normal length at the time of the removal of the dressing, and the lady walks to-day with the least perceptible halt.

I claim no originality for this mode of treating this kind of fracture, as the plan was fully set forth and illustrated by a wood cut, in a late number of the *American Journal of Medical Sciences*.

I venture to ask, may it not be more the inability of the surgeon to maintain the fragments in coaptation —as Prof. Gross states that no means yet known to the profession accomplishes this perfectly — rather than a physiological impossibility for the parts to unite by bony union, as claimed by Sir Astley Cooper? Had I followed that great master in the treatment of my patient, she would have undoubtedly become a cripple, and gone halting the balance of her life.

I do not think it possible to have had as good results follow the first appliance in this case. If by this report I shall induce the members of this Society to investigate the subject, and prove it good or bad, my object will have been fully accomplished.

